# Design and simulation of a gripper structure of cluster tomato based on manual picking behavior

**DOI:** 10.3389/fpls.2022.974456

**Published:** 2022-08-29

**Authors:** Yifeng Zheng, Jie Pi, Tiezheng Guo, Lei Xu, Jun Liu, Jie Kong

**Affiliations:** ^1^Key Laboratory of Protected Agriculture Engineering in the Middle and Lower Reaches of Yangtze River, Ministry of Agriculture, Institute of Agricultural Facilities and Equipment, Jiangsu Academy of Agricultural Sciences, Nanjing, China; ^2^Laboratory of Bionic Robot, Nanjing Institute of Technology, Nanjing, China

**Keywords:** cluster tomato, imitate human hands, picking behavior, gripper, finite element simulation

## Abstract

Picking robot technology plays an important role in the rapid promotion of precision agriculture. The development of a successful robot gripper is critical for the final promotion and industrialization of the tomato picking robot. This paper investigated the cluster tomato picking strategy and the gripper structure design based on this strategy to address the problem of poor adaptability of the existing gripper design in the cluster tomato picking scene. Starting from the research on the behavior of artificially picking cluster tomatoes, the grasping method, finger structure parameters and picking movement pattern of the human hand are analyzed. The evaluation criteria of the gripper are summarized, a simplified mathematical model of the gripper is established, and the picking strategy under the model of the gripper is proposed. Furthermore, according to the simplified gripper model, a rigid-flexible coupling gripper structure is designed, and the gripping simulation analysis is carried out. According to the simulation results, the gripper can smoothly grab medium and large tomatoes with diameter of 65∼95 mm. The peak force and fluctuation force of tomato with different sizes during grasping were less than the tomato’s minimum damage force. The gripper has adaptability and stability characteristics, providing technical support for gripper manufacturing and the construction of a picking system for a tomato picking robot.

## Introduction

Tomato is an important vegetable crop with an annual global production of more than 182 million tons and a planting area of more than 5.05 million hectares. China is a big country of fresh and processed tomatoes. The tomato cultivation area has exceeded 1.1 million hectares and the annual output is 65.15 million tons (Huajing Intelligence Network, 2021), of which the greenhouse tomato area is about 642,466 hectares (YMT Research Institute, 2022). In the entire tomato production chain, the most time-consuming and labor-intensive part is the picking link, whose labor demand accounts for more than 50% of the entire planting and production process (Ren et al., 2020). For a long time, there are problems such as high labor cost, low efficiency, and heavy workload.

In recent years, with the advancement of visual recognition and intelligent control technology, the completion of tomato harvesting by robots has become a new development trend (Wu et al., 2020). As the terminal part of the interaction between the picking robot and the tomato, the gripper has a significant impact on the working efficiency of the robot and the fruit damage rate. Therefore, a properly designed gripper is the key problem that the tomato picking robot needs to solve urgently. Under the premise of ensuring stable clamping, the damage to the tomato fruit is reduced, and the smooth grasping is realized.

At this stage, the research on tomato picking robot gripper can be divided into three categories: rigid gripper, flexible gripper and rigid-flexible coupled gripper. The research and application of rigid gripper in tomato picking has achieved many achievements. It has the advantages of large grasping force, high precision and fast speed. However, there are problems such as poor flexibility and weak adaptability, and the complex structure and insufficient flexibility can easily cause damage to the fruit epidermis, resulting in a low success rate of picking. Chih-Hsing Liu’s team at National Cheng Kung University designed a two-fingered tomato gripper with sensory feedback. It can grab tomatoes with a size of 50∼140 mm, and the maximum payload is 2.5 kg (Liu et al., 2019). Hiroaki Yaguchi’s team at the University of Tokyo designed a three-finger rigid gripper that rotates along a central axis to pick tomatoes. However, because the opening of the gripper is too large, it is often hindered by tomato vines, and the picking success rate is only 62.2% (Yaguchi et al., 2016). Naoshi Kondo’s team at Kyoto University developed a rigid tip for picking tomato clusters (Fujinaga et al., 2021), which was too bulky to perform actual picking operations. The picking success rate is only 50%. Chao Ji’s team at China Agricultural University developed a rigid end effector for shearing and gripping tomato stems (Ji et al., 2013). The short tomato stalk caused visual recognition difficulties. The picking time of a single fruit was 37.2 s, and the sharp blade was easy to damage the fruit.

The finger structure of the flexible gripper obtains infinite degrees of freedom through continuous deformation when interacting with the fruit, replacing the joints and connecting rods of the rigid gripper. It has obvious advantages when grasping soft and crisp tomato fruit. The Kehong Zhou team of Jiangsu University (Zhou et al., 2022) and the Asiwan Kultongkham team of King Mongkut’s University of Technology Thonburi (Kultongkham et al., 2021) designed a tomato-picking soft gripper based on fluid elastic actuation, which grasps the tomato by inflating and deflating. However, when faced with the problem of tomato stacking, due to the large size of the flexible fingers, it is not easy to penetrate into the tomato gap. The fin-ray effect-based gripper designed by Khaled Elgeneidy’s team at University of Lincoln has been gradually commercialized (Elgeneidy et al., 2019). It is universal for picking tomatoes of different sizes, but is prone to slippage when picking tomatoes with smooth surfaces. Vito Cacucciolo’s team at the University of Electro-Communications developed a flexible gripper based on electroadhesion and dielectric elastomers (Cacucciolo et al., 2019), weighing only 0.015 N. Small cherry tomatoes can be grasped, and the gripping force is more dependent on the grasping posture. Therefore, most of the tomato picking flexible grippers currently developed are more suitable for specific single or small tomato scenarios.

Rigid-flexible coupling gripper is an emerging research direction in agricultural picking. Scholars combine flexible and rigid structures to take into account the advantages of flexibility and rigidity. At present, the form of rigid-flexible coupling is mostly a combination of flexible suction cups and rigid fingers (Liu et al., 2007; Jun et al., 2021). This type of end effector adopts multiple sets of driving systems and structures, which increases the complexity of the control system, the production cost and the risk of fruit damage. The advantages of rigidity and flexibility are not fully exploited, and further in-depth research is urgently needed.

To sum up, the structural design of various tomato picking grippers needs to be further studied, which is rooted in two major difficulties. First, the tomatoes are suspended in clusters of 3–4. There are situations where tomatoes are stacked on each other, handles and leaves block tomatoes, etc., which has a great negative impact on the picking success rate of robot grippers. Second, grippers are not suitable for realistic picking scenarios, and there is no scientific theoretical basis for support. The above two difficulties lead to the problem of clustered tomato picking still to be overcome. Therefore, this paper takes the clustered medium and large tomatoes in the greenhouse as the picking objects, and combines the tomato manual picking behavior with the design of the gripper structure. A rigid-flexible coupling gripper imitating human hand picking was designed, and picking strategy was formulated for the gripper. Through the simulation analysis of the gripper, the reliability of the design is verified, which provides a reference for the manufacture and application of the gripper for tomato picking.

## Manual picking behavior of clustered tomato

### Grasping methods of human hands

Generally speaking, humans can accurately and efficiently pick tomatoes of different sizes in an unstructured greenhouse environment through the coordination of the brain, eyes and hands, without causing damage to the tomatoes. From the perspective of bionics, tomato picking grippers can improve performance through bionic design based on human hand grasping technology, which requires a deep understanding of human picking behavior. Human hand grasping is a highly complex movement that requires brain and vision-guided coordination of multiple finger joints and muscles (Ma et al., 2019). Therefore, it is necessary to observe the habit of human grasping tomatoes, study the factors that affect the grasping action, and summarize the commonly used grasping methods. The above has important scientific value for the design of the gripper, the implementation of an effective grasping strategy, and the realization of efficient, accurate and non-destructive picking.

Scholars have done part of the research on how the human hand grasps objects. Bullock et al. (2013) found that the most commonly used type of grip is partial wrapping. Lee and Jung found that the properties of the grasped object have a significant impact on the choice of grasping type, and the shape of the object can define the type of hand posture and limit the potential contact position of the hand (Lee and Jung, 2014). Feix et al. (2014) found that “grip” often applies for large and heavy objects, “pinch” always works for small and light objects. However, previous research work has not addressed the problem of clustered tomato picking. For example, there is little reference information on the relationship between the pose and geometric features of tomato fruits and the selection of human hand grip types during harvesting. In this paper, the method of experimental analysis is used to study the influencing factors of the selection of grasping methods when picking tomatoes manually.

### The experiment of picking tomatoes by hand

The experiment was carried out in the Institute of Agricultural Facilities and Equipment, Jiangsu Academy of Agricultural Sciences in January 2022. In the greenhouse where Jiangshu No. 1 tomato variety (medium and large tomato) was grown, 45 tomato crops in 3 columns were randomly selected and divided into 5 groups. 40 ripe tomatoes of different sizes and positions were randomly picked from each group. Five professional growers of different palm sizes participated in the experiment, and each participant naturally picked 40 tomatoes for analysis. During testing, greenhouse ambient temperature: 20 ± 2°C; relative humidity: 50–65%. Most robots usually adjust the pose to be directly under the fruit, move up the stem and grasp the fruit. The picking work is done by rotating and twisting the stem of the fruit through the degree of freedom at the end of the robotic arm. Therefore, when picking tomatoes, each participant is required to face the fruit. The right hand grabs and twists the stem from directly under the tomato. Photographs and videos were taken of each participant’s grasp of the tomato and the twisting of the stem.

### Picking experiment results

#### Tomato geometry and mass

**FIGURE 1:**
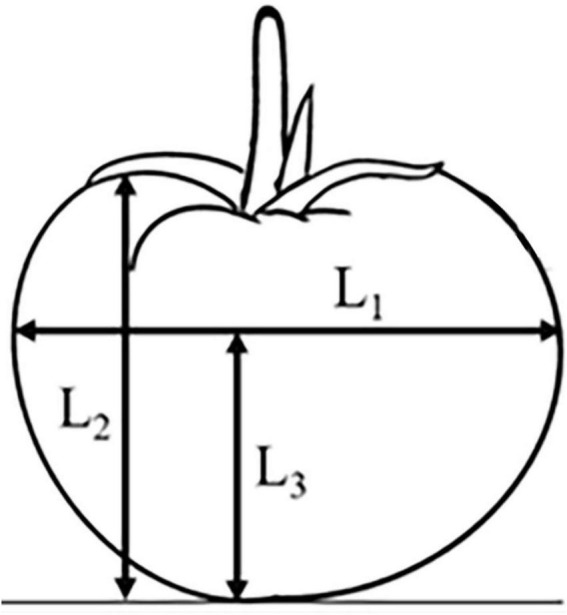
Diagram of tomato size.

Use a vernier caliper to measure the transverse diameter *L*_1_, longitudinal diameter *L*_2_ and transverse diameter height *L*_3_ of 200 tomatoes. The specific measurement positions are shown in Figure 1. The mass m of tomatoes was measured with an electronic scale JA5001 (Puchun, Shanghai, China).

The measurement results are shown in Table 1. The lateral diameter *L*_1_ of the tomato ranges from 66.4 to 92.7 mm, the longitudinal diameter *L*_2_ ranges from 52.6 to 81.3 mm, the height *L*_3_ of the lateral diameter ranges from 35.1 to 62.7 mm, and the tomato mass ranges from 152.7 to 378.2 g. Among them, tomatoes with a size of 75∼90 mm account for a large proportion, which proves that Jiangshu No. 1 is a medium and large tomato variety.

**TABLE 1:** Test results of geometry and mass.

Size/(mm)	Ratio/(%)	Max quality/(g)	Max transverse diameter height/(mm)
65∼70	1%	179.3	38.8
70∼75	6.5%	210.6	42.7
75∼80	36%	290.4	45.8
80∼85	31.5%	305.2	50.5
85∼90	22%	370.7	58.4
90∼95	3%	400.4	62.7

Size, lateral diameter of the tomatoes.

Ratio, proportion of tomatoes in this sze range to the total.

Max quality, maximum quality of a single tomato in this size range.

Max transverse diameter height, maximum transverse diameter height of a single tomato in this size range.

#### The types of human hand grasping

**FIGURE 2:**
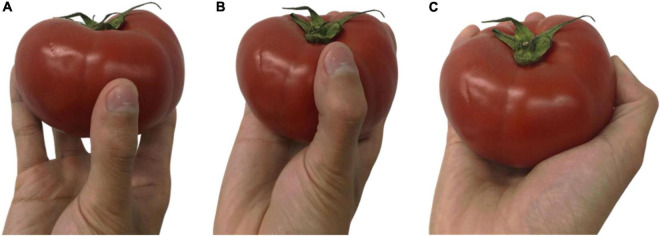
Human hand holding type of tomato picking **(A)** fingertip pinch type; **(B)** semi-enclosed grip type for fingers and palms; **(C)** full coverage grip type.

Referring to the statistical method of scholar Li et al. (2019), the experimental results were classified and counted. After the statistics of the experimental results, it was found that the tomato grasping types of each participant can be defined as three types: Fingertip pinch type, Semi-enclosed grip type for fingers and palms, and Full coverage grip type, corresponding to Figure 2.

**FIGURE 3:**
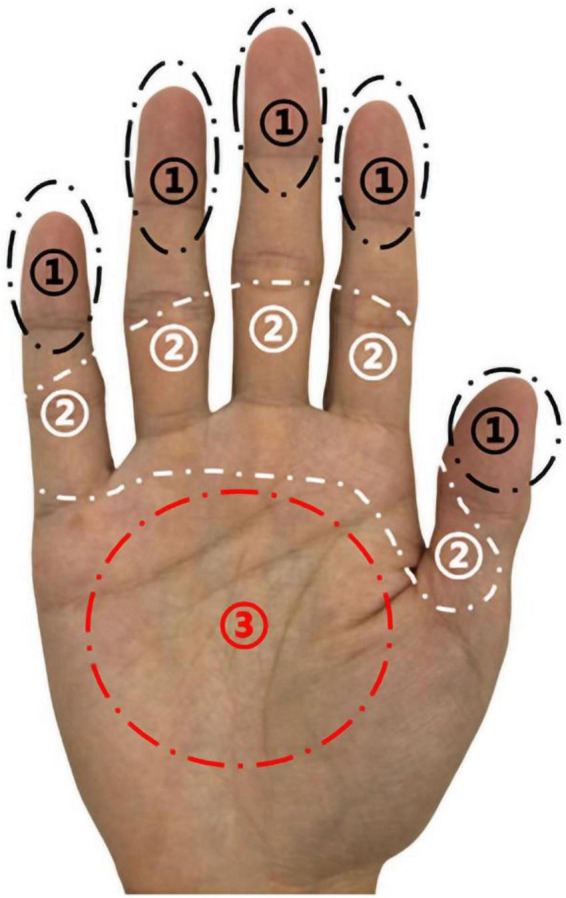
Schematic diagram of palm area division.

The palm during grasping is divided into three areas, the number ➀ is the fingertip area, the number ➁ is the root area of the finger, and the number ➂ is the palm area, as shown in Figure 3. Fingertip pinch type holds the tomato through the fingertips of 5 fingers (area ➀), with the thumb usually facing the other fingers. In the semi-enclosed grip type, the tomato is covered by the two contact areas (regions ➀ and ➁) of the fingertip and finger root, and is in contact with a small part of the palm. Full coverage grip type is covered by the fingertips, finger roots, and the full area of the palm (area ➀➁➂).

The grasp type statistics of 200 tomatoes are shown in Table 2. Fingertip pinch type and Full coverage grip type account for less, and most tomatoes are picked by Semi-enclosed grip type for fingers and palms. According to the comparison of the tomato transverse diameter, it was found that among the 20 tomatoes picked by pinching, 75% of the tomatoes were smaller than 75 mm, which proved that the participants usually picked tomatoes with smaller sizes by pinch grip. The 10 tomatoes picked by the full coverage grip were over 90 mm in diameter, proving that extra-large tomatoes require a greater grip. Semi-enclosed grasping with fingers and palms is the most popular grasping method. The transverse diameter of 167 tomatoes is in the range of 75–90 mm, which is the common size of Jiangshu No. 1. It can be determined that the semi-enclosed grip type is suitable for most Jiangshu No. 1 tomatoes.

**TABLE 2:** Statistics for tomato grip types.

	Fingertip pinch	Semi-enclosed grip type for fingers and palms	Full coverage grip
Quantity	20	170	10
Ratio	10%	85%	5%

**FIGURE 4:**
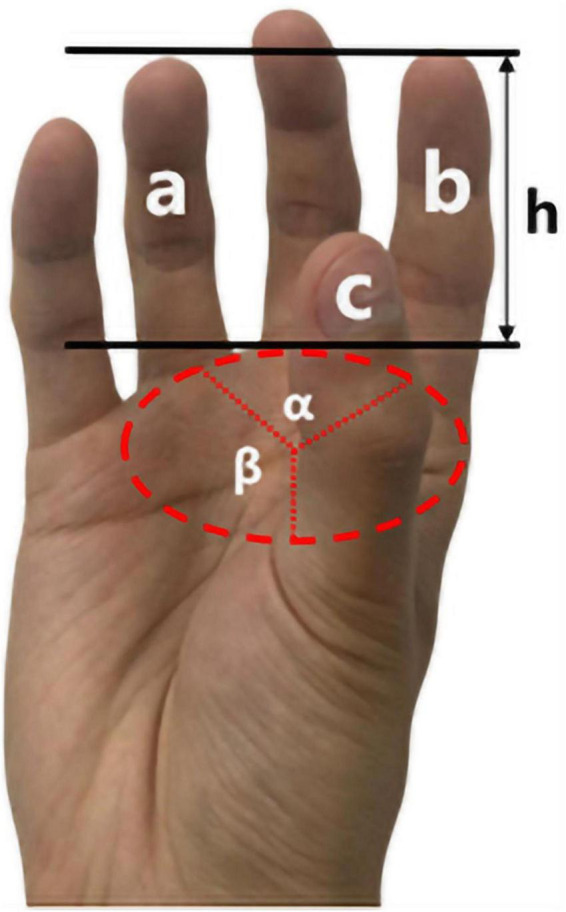
Semi-enclosed grip type for fingers and palms.

After further research, it was found that not all fingers exerted a grasping force when adopting the Semi-enclosed grip type of tomato picking. The fingers that actually exert the grip force are the ring finger a, the index finger b, and the thumb c. In order to measure the angle formed between the three fingers, a circular white paper is placed between the fingers, the position of the three fingers is marked, and the angle formed by the three fingers is measured with a protractor. As shown in Figure 4, in the circular area (red circle) formed by the three fingers, the angle between the two fingers is not a uniform 120°. The angle between the ring finger a and the index finger b is about 90°, and the angle between the ring finger a and the thumb c is about 135°. At the same time, the sizes of the three fingers a, b, and c are different, but after placing the white paper over the fingertips, it is found that the fingertips are on the same horizontal plane. The fingers do not cover the entire contour of the tomato, the height of the fingers only exceeds the height of the largest transverse diameter. The correctness of this view was confirmed by comparing the size of the a and b fingers of the participants and the size of the tomato arc. In addition, when the fingers exert force on the tomato, the rigid phalanx mainly ensures the stability of grasping, supplemented by the soft finger pad to cushion and protect the tomato. Therefore, when designing the gripper for tomato picking, it will adopt a rigid three-finger structure and wrap a layer of flexible material on the outside to simulate the characteristics of the rigid-flexible coupling of fingers. The three fingers are of the same size, and the length is not greater than the height of the largest transverse diameter of the tomato. The radian imitates the posture of human fingers, after measuring with the radian ruler, it is determined to be 10∼20°. The angles formed by the three fingers are set to 90° and 135°, respectively.

#### Manual picking strategy

The way of manually separating the tomato from the stem in the recorded video was analyzed. Before picking tomatoes, the experimenter rotated the tomato at a certain angle, so that the end of the stem was torn. Then a pulling action (instantaneous acceleration along the axis of the stem) is applied to the tomato, the pulling distance is about 3∼5 cm, and the tomato can be successfully separated from the stem. Unlike the robotic arm, the maximum rotation angle of the human hand is 180°. Therefore, it is possible to estimate the angular range of the rotation just by looking at the number of rotations and the stopping position. The statistical results of the rotation angle are shown in Table 3. The number of tomatoes with a rotation angle of less than 180° and between 180° and 360° was less, and only accounted for 22.5% of the total. Most tomatoes were spun more than one turn, somewhere between one turn and one and a half turns.

**TABLE 3:** Rotation angle before tomato separation.

	α ≤ 180°	180° < α ≤ 360°	360° < α ≤ 540°
Quantity	12	33	155
Ratio	6%	16.5%	77.5%

After in-depth research, it was found that tomatoes with a rotation angle of less than 180° usually fell off during the rotation. Such tomatoes are in an overripe state, some have cracked, have missed the best picking time, and are of poor quality. Tomatoes rotated between 180° and 360° are fully ripe. Although the quality of these tomatoes is at its best, it is no longer suitable for short-term storage. Tomatoes with a rotation angle between 360° and 540° are usually rotated twice (360°) and pulled without falling off. After continuing to rotate once (540°) and pulling, it can fall off the stem. Such tomatoes are just ripe and can be stored for a short period of time after picking, and are in the best picking state. Therefore, when picking tomatoes, make sure that the tomatoes are in the best picking condition. The rotation angle of the gripper is between 360° and 540°, which can ensure that the tomato can be smoothly separated from the stem.

The pulling action is the most vulnerable stage to damage the tomato, which will generate an instantaneous acceleration and force on the tomato. If the force is too high, the tomato will be damaged. In order to obtain the pulling force required for the pulling action and provide a reference for the design of the gripper picking strategy, 50 tomatoes in the best picking state were randomly selected for the pulling force experiment. The tomato and the dynamometer are fixed by a string. After the tomato rotates 360°, a momentary pulling action is applied from the end of the dynamometer to read the reading of the dynamometer. The results show that the average value of the instantaneous tensile force is about 11.66 N, and the maximum instantaneous tensile force reaches 15.29 N.

## Design of the picking gripper

### Evaluation criteria for grippers

After conducting literature research on the design of picking robot grippers, the evaluation criteria for gripper design for cluster tomato picking are summarized as follows:

(1) Picking objects (Navas et al., 2021): When performing the grasping task, the fingers generate a direct interaction force with the fruit. The fit between the shape and size of the fruit and the structure of the fingers is one of the most critical reference criteria for the rationality of the design.

(2) Gripper size (Shintake et al., 2018): The structure of the gripper is divided into a finger part and a fixed component part. The overall structure should follow the principle of volume minimization. Achieve picking tasks with the most compact structure and minimal workspace. The finger part should follow the principle of thin thickness and small volume. In the face of the fruit growing in clusters, the finger can penetrate into the crevice between the vine and the fruit.

(3) Lifetime (Chowdhary et al., 2019): This parameter is the number of days that the gripper can remain in normal operation before it malfunctions or exhibits a changed movement pattern. When the gripper is in use, it will be continuously loaded and open and closed, which wears the material of the structure. The lifetime is an important indicator for evaluating the gripper.

Based on the above evaluation criteria, the structure and tomato characteristics should be considered when designing the gripper. Based on the design criteria of thin fingers and simple and easy-to-control structure, the tomato grasping is realized with a light structure and an exquisite picking method.

### Design of gripper structure

**FIGURE 5:**
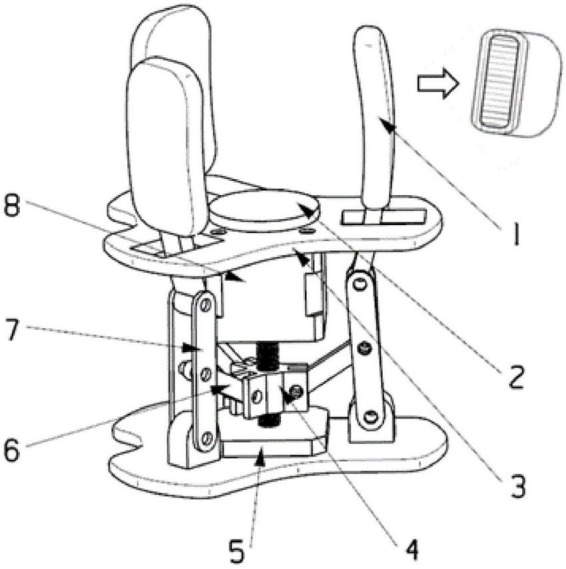
Gripper structure design (1. Finger 2. Flexible finger pad 3. Limit flange 4. Slider 5. Support flange 6. Limit link 7. Support link 8. Screw motor).

The structure design of the gripper is carried out according to the semi-enclosed grasping type of the fingers and palms. As shown in Figure 5, the gripper is composed of a fixed component, a driving mechanism and fingers. The fixed assembly includes a limit flange and a support flange. The limit flange is used to install the screw motor and limit the working stroke of the fingers. The bottom of the support flange is assembled with the robotic arm. The tetrahedral structure with raised surface plays the role of protection and limit. The drive mechanism consists of three limit links, three support links, a slider and a screw motor. The overall use of the under-driven structure. The slider moves up and down on the screw rod to drive the limit link and the support link to rotate, thereby driving the fingers to open and close. The number of fingers is 3, the angles formed by the three fingers are 90° and 135°, the finger length is 60 mm, and the arc is set to 15°. A layer of flexible material with a thickness of 2 mm is wrapped around the finger, as shown in the cross-sectional view of the finger in Figure 5. In addition, a layer of flexible material is installed on the limit flange to simulate the contact method of a small part of the palm and the tomato when grasping the tomato. The flexible material cushions tomatoes when the gripper is picking.

In addition, for the controllability of the gripper, 4 piezoelectric thin film sensors are added. The accuracy of the sensor can reach ± 0.1 N. When making flexible finger pads and finger cots, thin film sensors are embedded in uncured colloid. After the colloid is solidified and formed, the sensor and the colloid form an integral body. Relying on 4 force feedback information, the gripper can form a reliable closed-loop control system.

**FIGURE 6:**
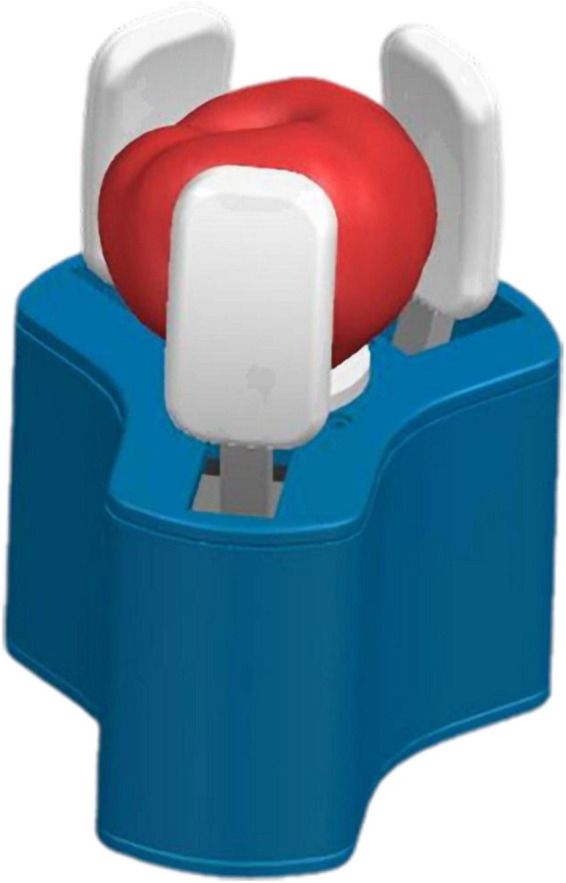
Gripper grasps tomato.

After analyzing the actual scene of tomato planting, a protective sleeve was added between the support flange and the limit flange, as shown in Figure 6. On the one hand, the greenhouse environment is hot and humid. The connecting rod and motor are prone to corrosion and fracture after long-term operation. On the other hand, tomato vines are entwined. During the picking task, the branches and leaves are easy to penetrate into the gap of the gripper structure, causing the mechanism to jam and affecting the lifetime of the gripper. Therefore, adding a protective sleeve cannot only prolong the lifetime of the gripper, but also improve the success rate of picking.

### Key parameters of the gripper

**FIGURE 7:**
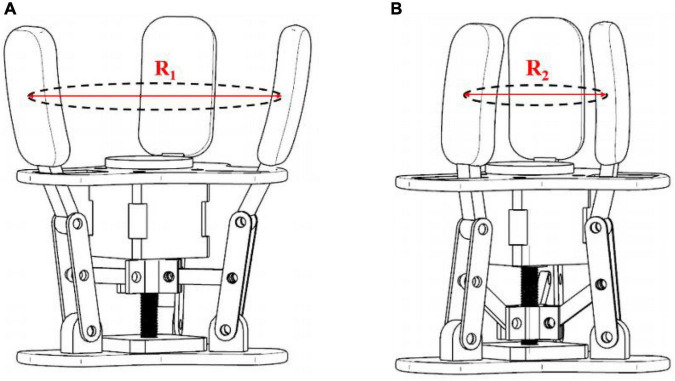
Limit space for gripper opening and closing **(A)** limit opening size of fingers; **(B)** limit closing size of fingers.

With reference to the tomato size measurement and basic design parameters, the design dimensions and structural materials of each part of the gripper are further determined. The 200 tomato varieties of Jiangshu No. 1 have a maximum lateral diameter of 92.7 mm and a minimum diameter of 66.4 mm. Therefore, in the limit space where the jaws are opened and closed as shown in Figure 7, when the fingers are in the state of limit opening, the diameter *R*_1_ of the middle of the fingers should be larger than the maximum transverse diameter of the tomato. The diameter *R*_2_ should be smaller than the minimum transverse diameter of the tomato in the state of extreme closed fingers. Consider that the tomato needs to go through the opening formed by the three fingertips first when picking. Moreover, the fingers have a certain radian, resulting in a small diameter of the circle formed at the tip of the three fingers, so set *R*_1_ to 110 mm. Set *R*_2_ to 60 mm to ensure the grip effect of smaller size tomatoes, and control the stroke of fingers and sliders within a certain range.

**FIGURE 8:**
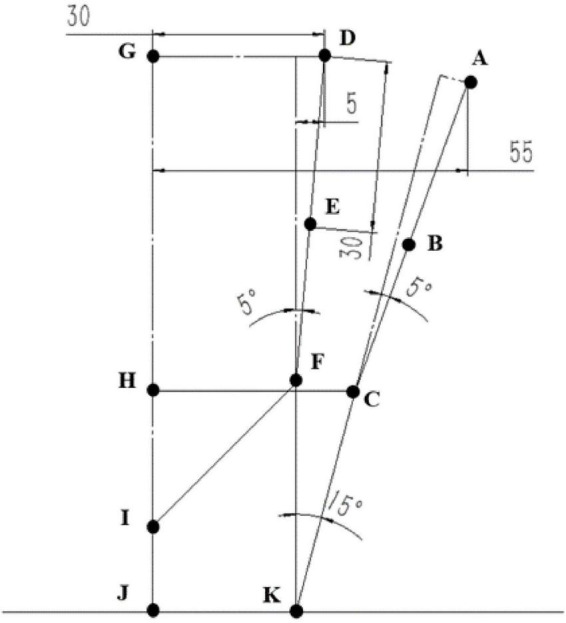
Simplified mathematical model of gripper.

A simplified mathematical model of the gripper is established as shown in Figure 8. The distance from point A and point D to the center line GH represents the radius of the circle formed by the middle of the finger when the finger is in the limit opening and closing state, which are 55 and 30 mm, respectively. AB = DE = 30 mm, representing half the length of the finger. According to the general selection of the stepper motor with less torque, the length of the support link to the center line JK is set to 25 mm. In order to ensure that the opening range of the gripper before picking is larger than the diameter of the tomato, a small inclination angle of 5° was set between the fingers and the support link. Set the rotation range of the support link to 15° when the fingers are in the limit tension state to ensure that the slider can be opened and closed by the fingers in a small stroke. After the above values are determined, the remaining connecting rod model length can be obtained: HJ = 39 mm, which represents the distance from the center of the slider to the bottom of the screw in the limit closed state. HC = FI = 35 mm, which represents the distance from the center of the slider to the center of the support rod. CK = FK = 40 mm, which represents the length of the support link. DF = AC = 58 mm, which represents the distance from the middle of the finger to the support link. HI = 24 mm, representing the stroke of the slider.

The structural parts of the gripper are made of nylon. Compared with resin materials, nylon materials have high temperature resistance, good toughness, high strength, and longer service life. Silicone was chosen as the flexible material covering the gripper fingers and over the flange for tensile strength and tear resistance. And it has strong adaptability to the greenhouse environment.

### Gripper picking strategy

**FIGURE 9:**
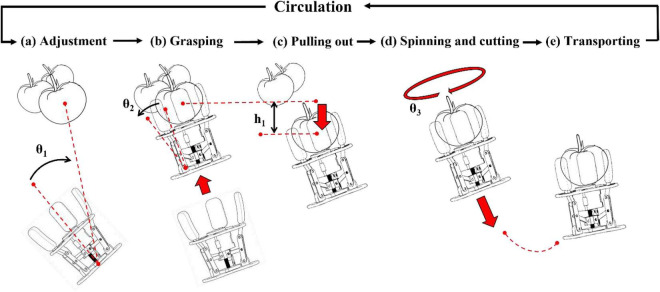
Tomato picking strategies.

Combined with the particularity of the clustered tomato hanging and the picking method imitating the human hand, a new tomato picking strategy as shown in Figure 9 was determined for the gripper. The strategy is divided into five steps, including adjusting, grasping, pulling out, Spinning and cutting, transporting. In step (a), the robot arm is guided to rotate by the vision system, and the gripper is adjusted to the pose as shown in the figure. This pose can keep the fingers of the gripper on the outside of the clustered tomatoes, avoiding the situation that the tomato positioning information may change when the gripper goes deep into the tomato gap. In step (b), after determining the position and posture, the gripper moves along the stalk collinearly, approaching the tomato until the tomato touches the flexible finger pad of the limit flange. The controller receives the force information fed back by the sensor in the flexible finger pad, and drives the stepper motor to close the finger until the flexible finger pad touches the tomato. At this time, the controller will receive the information feedback from the sensor in the finger cots, and stop the finger closing movement when the pressure reaches the minimum damage force of the tomato. In step (c), the gripper grasps the tomato and moves downward by a height h1 to ensure that subsequent operations are not affected by fruit stacking. In step (d), after the pull out stage is completed, the robotic arm drives the gripper to rotate 540°, so that the connection between the stem and the tomato is torn. A displacement with instantaneous acceleration is applied to the axis of the stem to complete the separation of tomato and stem. The displacement distance is about 5 cm, and the pulling force is about 15 N. Step (e) is the final transport stage, the gripper transports the picked tomatoes to the designated position, and the cycle continues from step (a).

## Gripper simulation experiment

### Force simulation experiment

#### Force analysis of tomato

When the operation of the gripper on the tomato enters the gripping stage, the contact area and force between the tomato and the finger will have a greater impact on its deformation. The finite element software ANSYS (ANSYS 2020R2) was used to simulate the force and deformation of tomato. Analyze the deformation of tomato when it is in contact with fingers of different widths and select the most suitable finger width. Referring to the research results of scholars (Li, 2011), the material properties of tomato are set as follows: elastic modulus: 0.762 Mpa, Poisson’s ratio: 0.45, stress intensity: 0.122 Mpa, density: 1,070 × 10^–9^kg/m^3^. Create a Static Structural module in ANSYS Workbench, add material properties to get the ANSYS model. After that, the mainstream tetrahedral element is used to divide the model into the network, and the element size is set to 1 mm, and a total of 72,708 nodes and 37,018 elements are divided.

**FIGURE 10:**
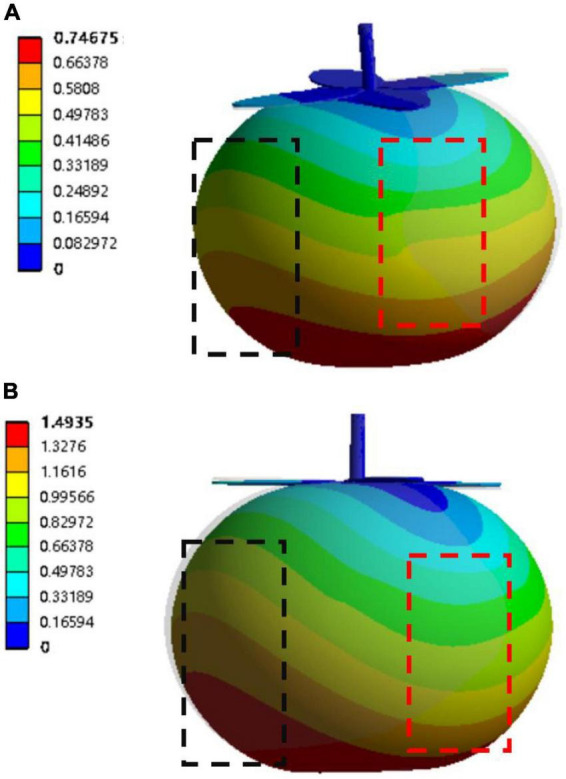
Tomato deformation simulation **(A)** 5 N load simulation; **(B)** 10 N load simulation.

The contact area between the finger and the tomato will expand as the width of the finger increases. When the finger width reaches 30 mm, the effective contact area with the tomato reaches the maximum. As the width of the fingers further increases, the curvature of the fingers can no longer match the curvature of the tomato. The increased width cannot form effective contact with the tomato and becomes an ineffective contact area. Moreover, fingers that are too wide may have a certain negative impact on the picking effect of clustered tomatoes. Therefore, the finger width of the gripper is divided into five categories: 10, 15, 20, 25, and 30 mm. Finger widths above 30 mm are not studied. According to the mesh size calculation, set the force area when the tomato is in contact with the finger. Taking a finger width of 30 mm as an example, the contact area between a single finger and a tomato is about 1,800 mm^2^. When selecting the force-bearing area, 30 unit surfaces are selected horizontally, and 60 unit surfaces are selected vertically. The total number of unit surfaces is about 1,800, which is consistent with the actual contact area to the greatest extent. Set the stem and leaf on the top of the tomato as a fixed support. Apply a load force of 5 and 10 N to the corresponding force area, respectively, with the direction inward, and solve the total deformation and displacement of the tomato. Figure 10 shows the tomato deformation simulation when a 30 mm finger exerts a force of 5 and 10 N on the tomato.

When the tomato is subjected to the load force exerted by the finger, as shown by the red dotted box area, the force-bearing part of the tomato deforms and displaces. At the same time, due to the extrusion of the force-bearing part, the other two sides and the lower part of the tomato experienced a large expansion displacement, as shown in the black dotted box area. Therefore, the maximum deformation of the tomato did not occur in the force-bearing part of the tomato, but occurred below the area on both sides of the force-bearing part, which was in line with the actual force of the tomato. The final simulation results are shown in Table 4. With the increase of the finger width and the expansion of the force-bearing area, the maximum deformation of the tomato and the displacement of the force-bearing area gradually decrease, which proves that the increase of the finger width has a certain protective effect on the tomato. The research on the physical properties of tomato according to the literature (Li et al., 2010) shows that the tomato is elastic deformation when the deformation amount is less than 1.5 mm, which will not affect the quality and storage time of the tomato. Therefore, a finger structure with a width of 30 mm was chosen. The force applied by a single finger to the tomato should be less than 10 N to ensure that the tomato is harvested without damage to the greatest extent.

**TABLE 4:** Statistics for tomato grip types.

Finger width (mm)	Force area (mm^2^)	Load (*N*)	Max deformation (mm)	Deformation of the stressed area (mm)
10	600	5	1.20	0.66∼0.90
		10	2.05	1.18∼1.67
15	900	5	1.07	0.59∼0.77
		10	1.91	1.07∼1.50
20	1,200	5	0.96	0.53∼0.69
		10	1.76	0.95∼1.37
25	1,500	5	0.84	0.48∼0.62
		10	1.62	0.89∼1.26
30	1,800	5	0.75	0.41∼0.58
		10	1.49	0.83∼1.16

#### Force analysis of single finger

In order to reduce the damage to the tomato caused by the instantaneous load when the finger is in contact with the tomato, a 2 mm thick silicone finger cover is added to the outside of the finger structure. In order to further verify the protection of the silicone finger cover to tomatoes, finite element force simulations were performed on the fingers with silicone finger cover and nylon fingers. According to the literature (He et al., 2019), the properties of the silicone material are set as follows: elastic modulus: 2.14 Mpa, Poisson’s ratio: 0.48, density: 1,200 kg/m^3^. According to literature (Guessasma and Beaugrand, 2019), the nylon material properties are set as follows: elastic modulus: 1.11 × 103 Mpa, Poisson’s ratio: 0.35, density: 1,140 kg/m^3^. The model uses tetrahedral elements for network division. The element size is set to 1 mm, and a total of 203,830 nodes and 126,875 elements are divided. A 5 N load is applied to the corresponding finger surface to simulate the force state of the finger.

**FIGURE 11:**
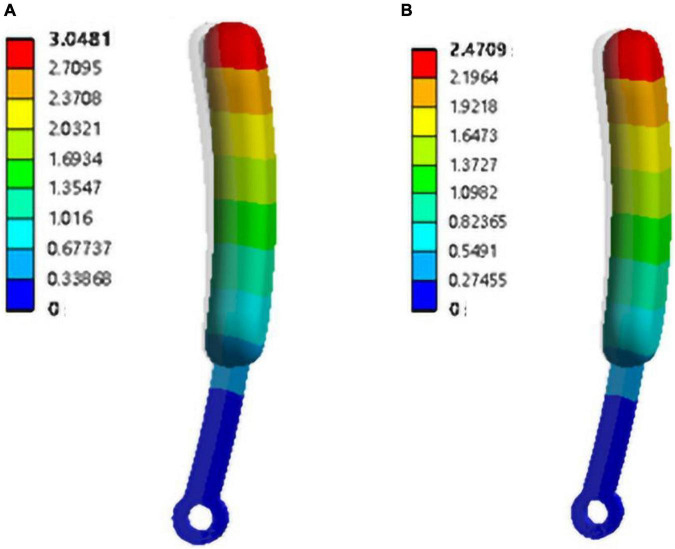
Finger deformation simulation **(A)** silicone covered finger; **(B)** nylon finger.

The simulation results are shown in Figure 11. The total displacement of the silicone-coated finger is about 3.05 mm, and the total displacement of the pure nylon finger is about 2.47 mm. The larger amount of finger deformation can provide a buffer for the tomato’s stress deformation, which proves that the silica gel material has a certain protective effect on the tomato.

### Motion state simulation experiment

**FIGURE 12:**
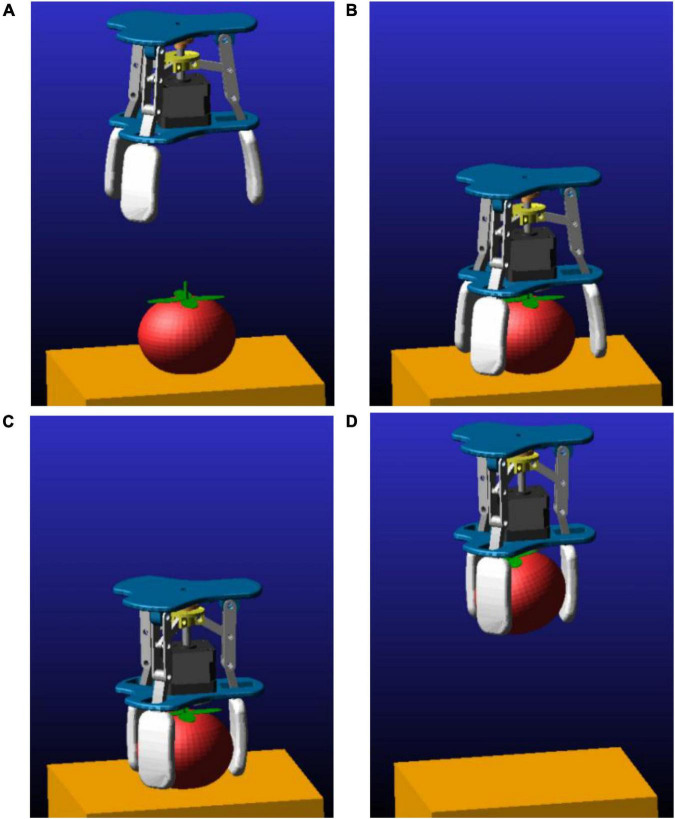
Gripper gripping state simulation **(A)** adjustment phase; **(B)** descending phase; **(C)** grasping phase; **(D)** uplift phase.

In order to verify the kinematic performance of the gripper, its motion state was simulated with Adams2020 software. Import the gripper and tomato models into the software. Set the rotating pair, the moving pair and the screw pair for the connecting rod, the slider and the screw. Adjust the jaw opening and closing speed by setting the screw torque. As shown in Figure 12, the four stages of the gripper grasping the tomato are simulated, namely adjustment, descent, grasping, and lifting. Analyze the gripping state of the gripper during this process.

#### Motion state simulation

Simulate the movement state of the gripper grasping a tomato with a transverse diameter of 80 mm. Set the tomato material properties as follows: Mass: 300 g. Elastic modulus: 0.762 Mpa. Poisson’s ratio: 0.45. Density: 1,070 × 10–9 kg/m^3^.

(1) Finger movement state

**FIGURE 13:**
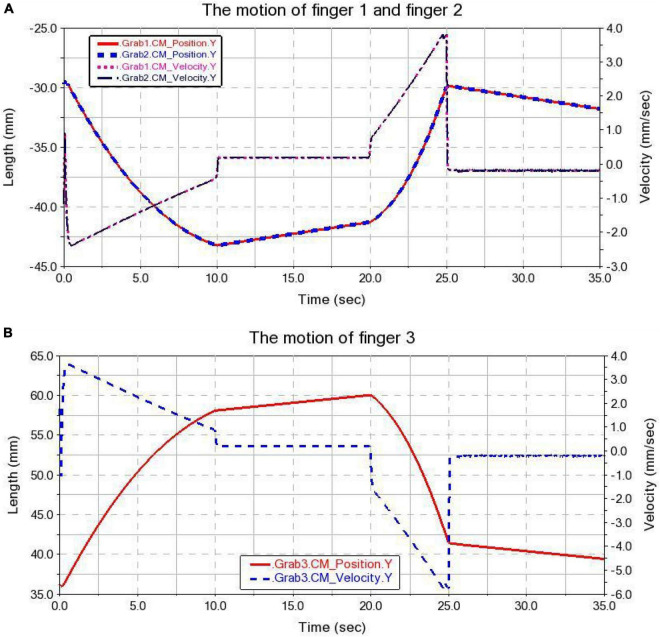
Finger movement **(A)** the motion of finger 1 and finger 2; **(B)** the motion of finger 3. (2) Slider motion state.

The displacement and velocity states during finger movement are shown in Figure 13. The displacement process of the three fingers is basically the same. In the interval of 0∼10 s, the finger is in the adjustment stage. From the semi-closed state (the finger is about 35 mm away from the center line) to the fully open state (the finger is about 55 mm away from the center line). Finger 1 and finger 2 are displaced by about 13 mm in the Y-axis direction, and finger 3 is displaced by about 22 mm. In the interval of 10–20 s, the finger is in a steady descent stage. In the 20–25 s interval, the gripper is in the grasping phase. The displacement of finger 1 and finger 2 is about 11 mm along the Y-axis direction, and the displacement of finger 3 is about 17.5 mm. At 25 s, there was a small fluctuation in the displacement velocity due to the finger touching the tomato. In the interval of 25–30 s, the finger is in a steady upward state. During the finger opening and closing movement, the overall displacement and velocity curves are smooth and stable. Comparing the simulation results with the simplified mathematical model above verifies the correctness and rationality of the design.

(2) Slider motion state

**FIGURE 14:**
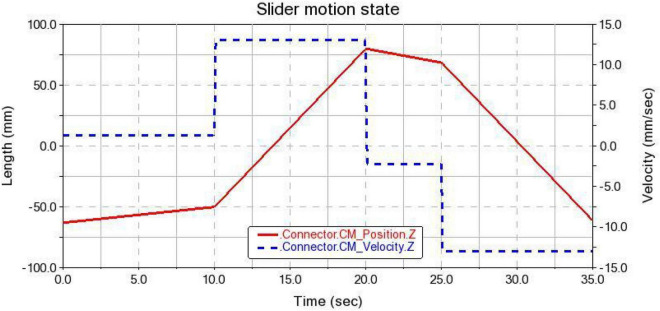
Slider motion status.

During the gripping process of the gripper, the motion state of the slider is shown in Figure 14. In the interval of 0∼10 s, the slider displacement is about 12.5 mm. In the interval of 20∼25 s, the displacement of the slider is about 10 mm. The slider displacement does not exceed the maximum stroke range, which is in line with the motion and design requirements.

#### Gripping state simulation

(1) Gripping and Movement Process Analysis

**FIGURE 15:**
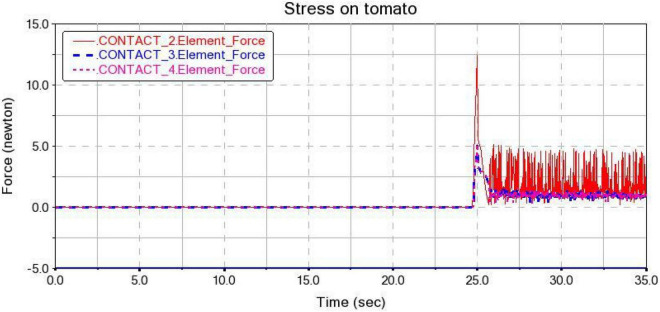
The force of tomato with a diameter of 80 mm.

Three tomato models with transverse diameters of 70, 80, and 90 mm were established. The mass was set to 200, 300, 400 g, respectively. A simulated tomato-grip gripping force test was performed. The force of tomato with a transverse diameter of 80 mm is shown in Figure 15. When the finger is in contact with the tomato, the instantaneous contact force reaches 12.5 N. The contact force drops rapidly within 1 s. During the subsequent lifting of the gripper, the force of the tomato has been fluctuating within the non-destructive force range of 5 N. Tomatoes with a diameter of 70 and 90 mm are subjected to similar forces. Moreover, during the entire tomato grasping process, no falling phenomenon occurred. Therefore, the gripper can protect the tomato from damage, and has strong stability and protection. This proves that the design of the gripper satisfies the requirements.

(2) Pulling Process Analysis

**FIGURE 16:**
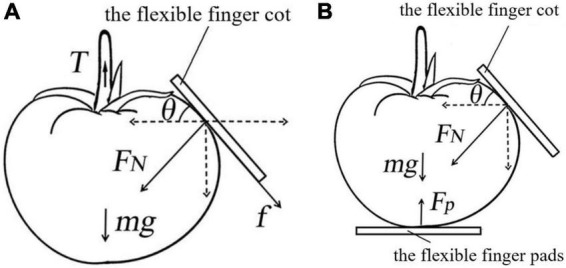
Force analysis of pulling process **(A)** pulling phase; **(B)** falling stage.

After the tomato is clamped, the force can be divided into two stages: the pulling stage and the falling stage. When the tomato enters the pulling stage, the force on the tomato includes the pulling force *T* of the stem on the tomato, the pressure *F*_*N*_ of the three fingers on the tomato, the friction force *f*_*p*_ between the tomato and the surface of the finger, and the gravity *mg* of the tomato, as shown in Figure 16A. Kinetic analysis was performed to obtain the conditions for the gripper to successfully separate the tomato from the stem:


(1) $$3\,\left(F_{\text{N}}\,\cos\theta +f_{\text{p}}\,\sin\theta \right)+m\,g\geq T$$

In the above formula, *f*_*p*_ = *μ_*s*_*, *F*_*N*_, *g* = 9.8 N/kg, *m* = 0.3 kg. θ is the inclination of the contact surface between the finger and the tomato. Set θ to 75° according to the finger arc. According to the literature (Zhou et al., 2022), the static friction coefficient *μ_*s*_* on the silicone pad is 1.34 N/N. According to the data obtained from the above experiments, the maximum instantaneous tensile force *T* is about 15.29 N. Bring in the formula to obtain *F_*N*_* ≥ 2.65 N, 3*F_*N*_* ≥ 7.95 N, indicating that the total force of the three fingers on the tomato is less than the minimum damage force of the tomato.

When the tomato enters the falling stage, the force on the tomato includes the pressure *F*_*N*_ of the three fingers, the supporting force *F*_*p*_ of the finger pad and the gravity *mg* of the tomato, as shown in Figure 16B. The kinematic analysis of the tomato at this time is carried out, and the force state formula is obtained:


(2) $$3\,F_{N}\,\cos\theta +m\,g=F_{p}$$

Substitute *F*_*N*_ = 2.65 N into formula (2) to obtain *F*_*p*_ = 5.00 N. Since *F*_*p*_ is less than the minimum damage force of the tomato, it proves that the gripper will not damage the tomato in the pulling stage and the falling stage.

## Conclusion and outlook

(1) This paper proposes a combination of tomato manual picking behavior and gripper design. Based on the artificial hand picking method, a rigid-flexible coupling gripper structure and picking strategy for Provence tomato picking are designed.

(2) According to the experimental results of hand-picking tomatoes, it is concluded that the lateral diameter *L*_1_ of tomatoes ranges from 66.4 to 92.7 mm. The longitudinal diameter *L*_2_ ranges from 52.6 to 81.3 mm. The transverse diameter and height *L*_3_ range from 35.1 to 62.7 mm. The tomato mass ranged from 152.7 to 378.2 g. Among the grasping methods of human hands, the most suitable picking hand type is Semi-enclosed grip type for fingers and palms. The in-depth study of the gripping method provides a scientific basis for the design of the gripper.

(3) Based on the evaluation criteria of the gripper, the three-finger structure of the rigid-flexible coupling gripper is designed. The overall use of the under-driven structure. The limit link and the support link are driven to rotate by the slider moving up and down on the screw rod. The prototype is based on the manual picking of the actual three fingers. The three fingers of the gripper are 90° and 135°, the finger length is 60 mm, and the arc is 15°. The finger is wrapped with a layer of 2 mm silicone material. The diameter of the middle part of the fingers is 60∼110 mm in the limit state of opening and closing. The picking strategy for the gripper was designed based on the way the tomato and stem were separated by hand. When the tomato and stem are separated, rotate the stem 540° and apply an instantaneous acceleration along the axis of the stem. Pull the distance about 5 cm, and separate the tomato from the stem.

(4) The force analysis and simulation of tomato was carried out. When the finger width is 30 mm, and the force exerted by a single finger on the tomato is less than 10 N, the tomato can be picked to the greatest extent without damage. The single-finger force analysis was carried out to prove the protective property of the silica gel material on tomato. Through the simulation of the gripping state of the gripper, it is verified that the motion state of the gripper meets the design requirements. The peak force and fluctuating force of tomato of different sizes were smaller than the minimum damage force of tomato.

In future research, multiple tomato varieties will be studied, and an index library of gripper design will be established according to the picking strategy in this paper, including the maximum opening, minimum damage force, maximum instantaneous pulling force, maximum gripping force, etc. Combined with the intelligent pressure sensor, while improving the picking speed, the mechanical damage rate is controlled to ensure the post-harvest quality. It will also focus on the research on the combination of tomato visual recognition positioning and picking strategy, and put the gripper into the experiment of the actual tomato picking scene.

## Data availability statement

The original contributions presented in this study are included in the article/Supplementary material, further inquiries can be directed to the corresponding author.

## Ethics statement

Written informed consent was obtained from the individual(s) for the publication of any potentially identifiable images or data included in this article.

## Author contributions

YZ: conceptualization, methodology, and writing—original draft preparation. JP: mechanical modeling, experiment design, and analysis. TG: writing—editing. LX: simulation and project administration. JL: funding acquisition and writing—reviewing. JK: kinetic analysis and experimental data collation. All authors have read and agreed to the published version of the manuscript.
