# Supplementary figures and images for: Design and simulation of a gripper structure of cluster tomato based on manual picking behavior
Source: Front Plant Sci. 2022 Aug 29;13:974456. doi: 10.3389/fpls.2022.974456 (PMC9465300; doi:10.3389/fpls.2022.974456)

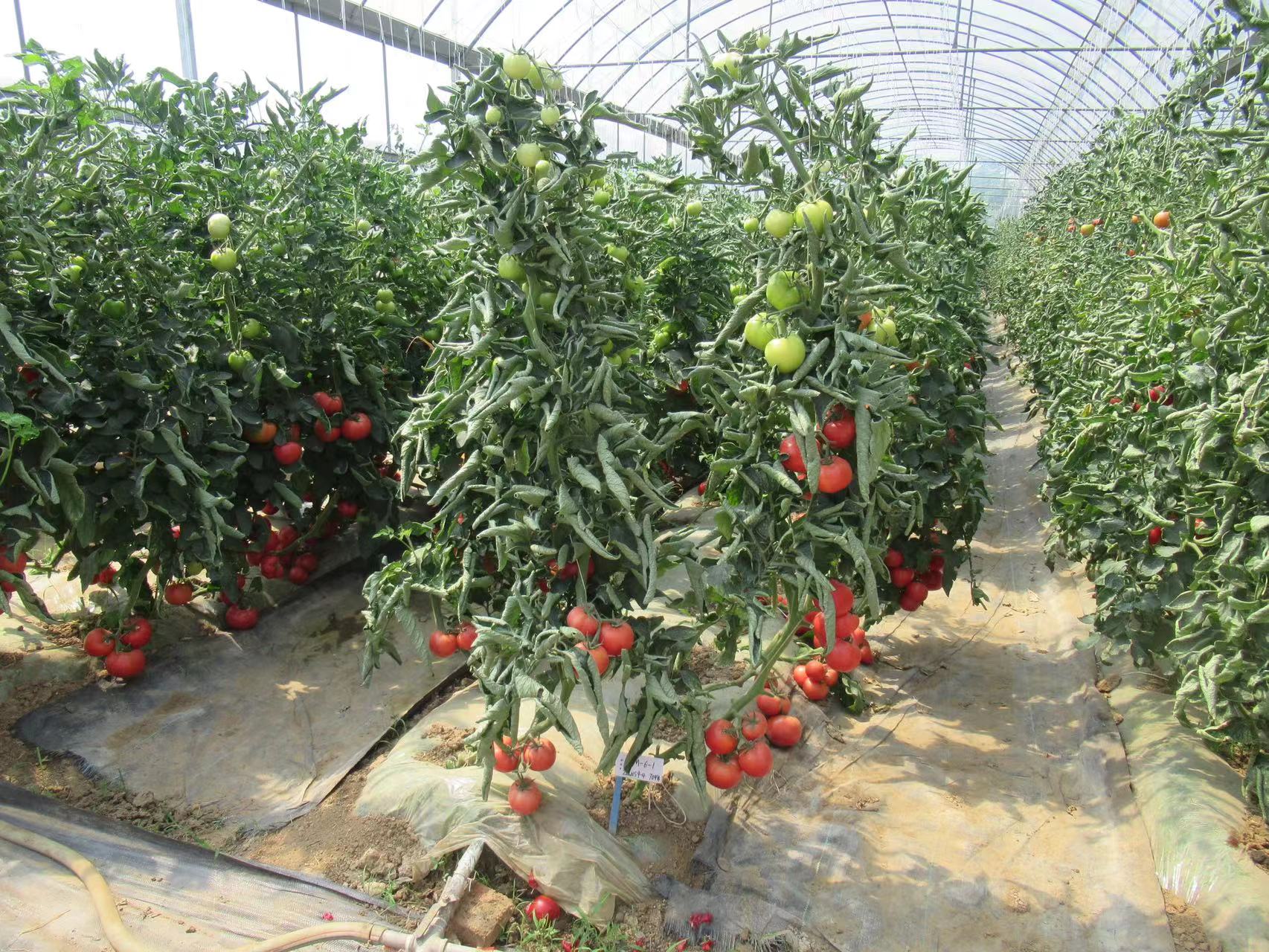

Supplement: Supplementary file 1 [file Data_Sheet_1.ZIP › Supplementary material/1.Facility tomato cultivation(soil cultivation).jpg]

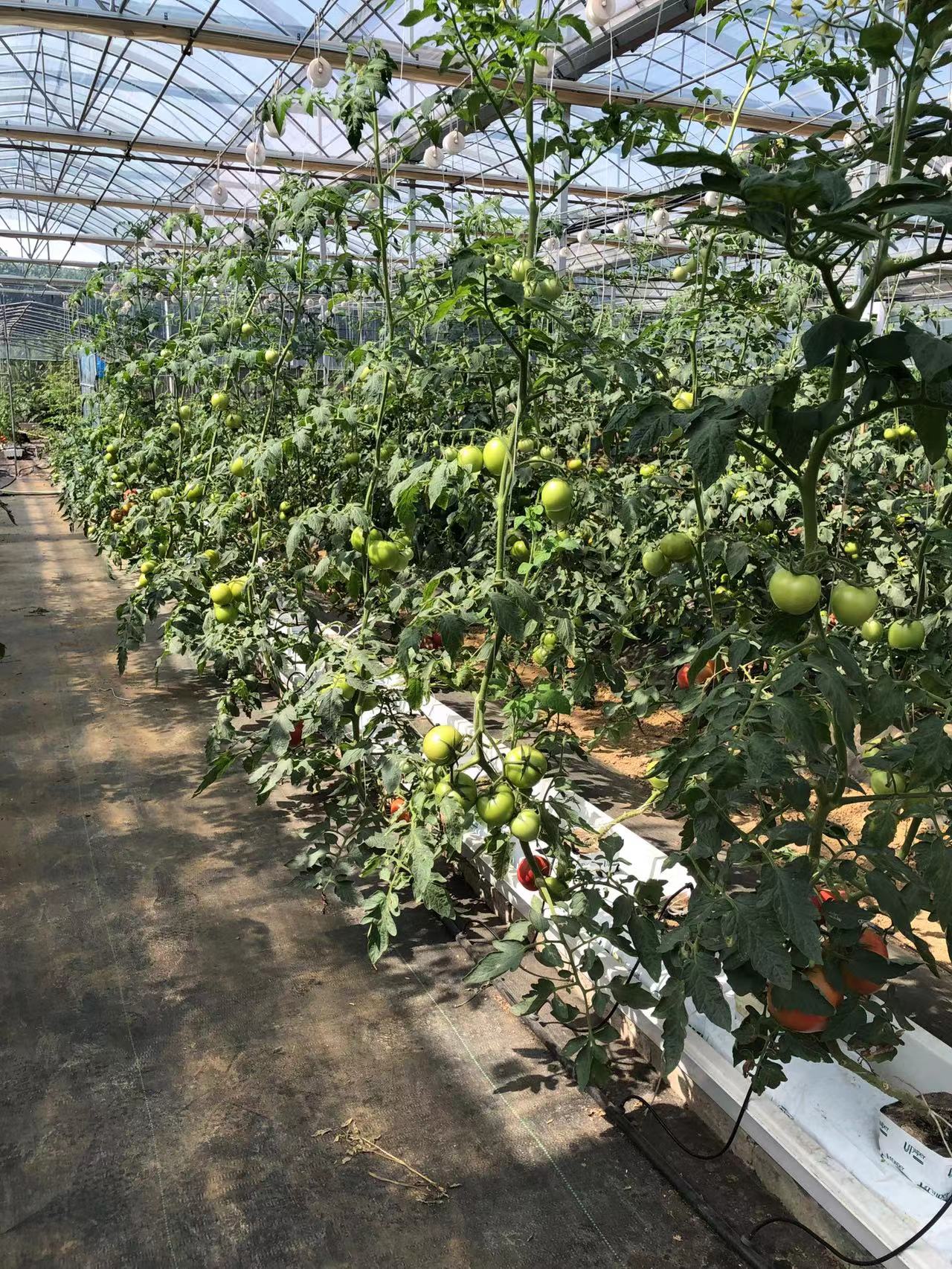

Supplement: Supplementary file 1 [file Data_Sheet_1.ZIP › Supplementary material/2. A bunch of tomatoes(soilless cultivation).jpg]

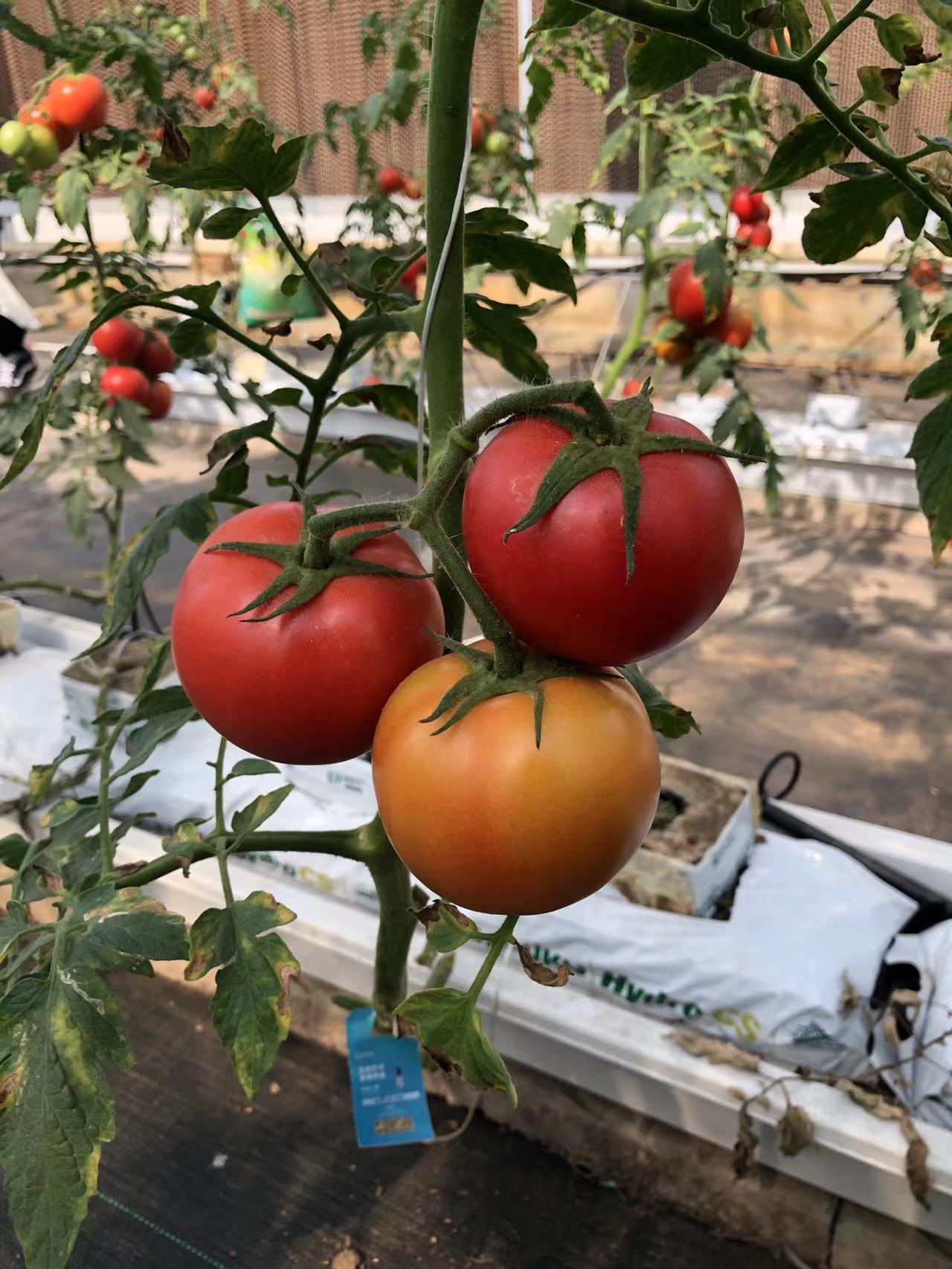

Supplement: Supplementary file 1 [file Data_Sheet_1.ZIP › Supplementary material/3. Clustered Tomato - 3 Fruits.jpg]

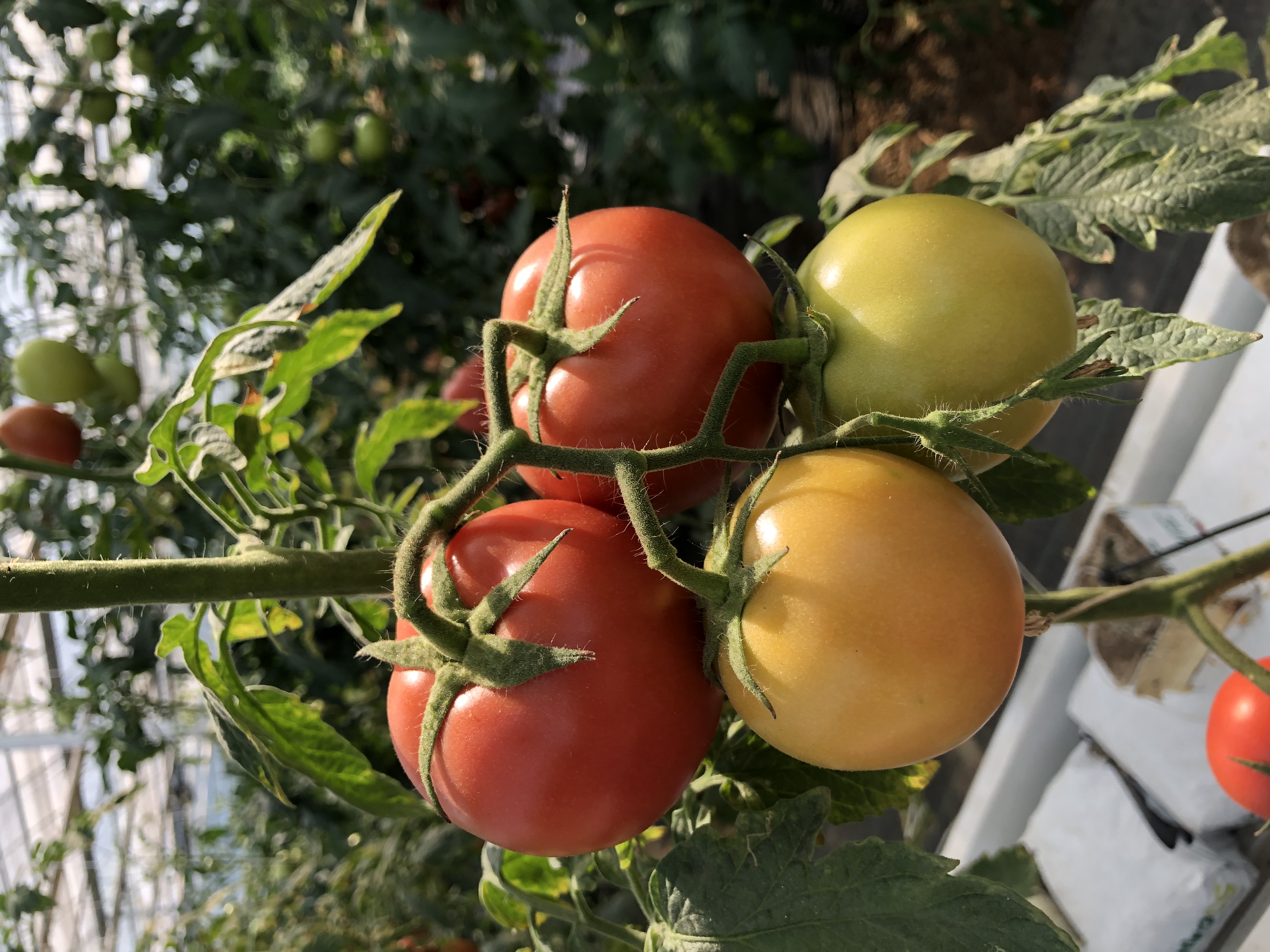

Supplement: Supplementary file 1 [file Data_Sheet_1.ZIP › Supplementary material/4. Clustered Tomato - 4 Fruits.jpg]

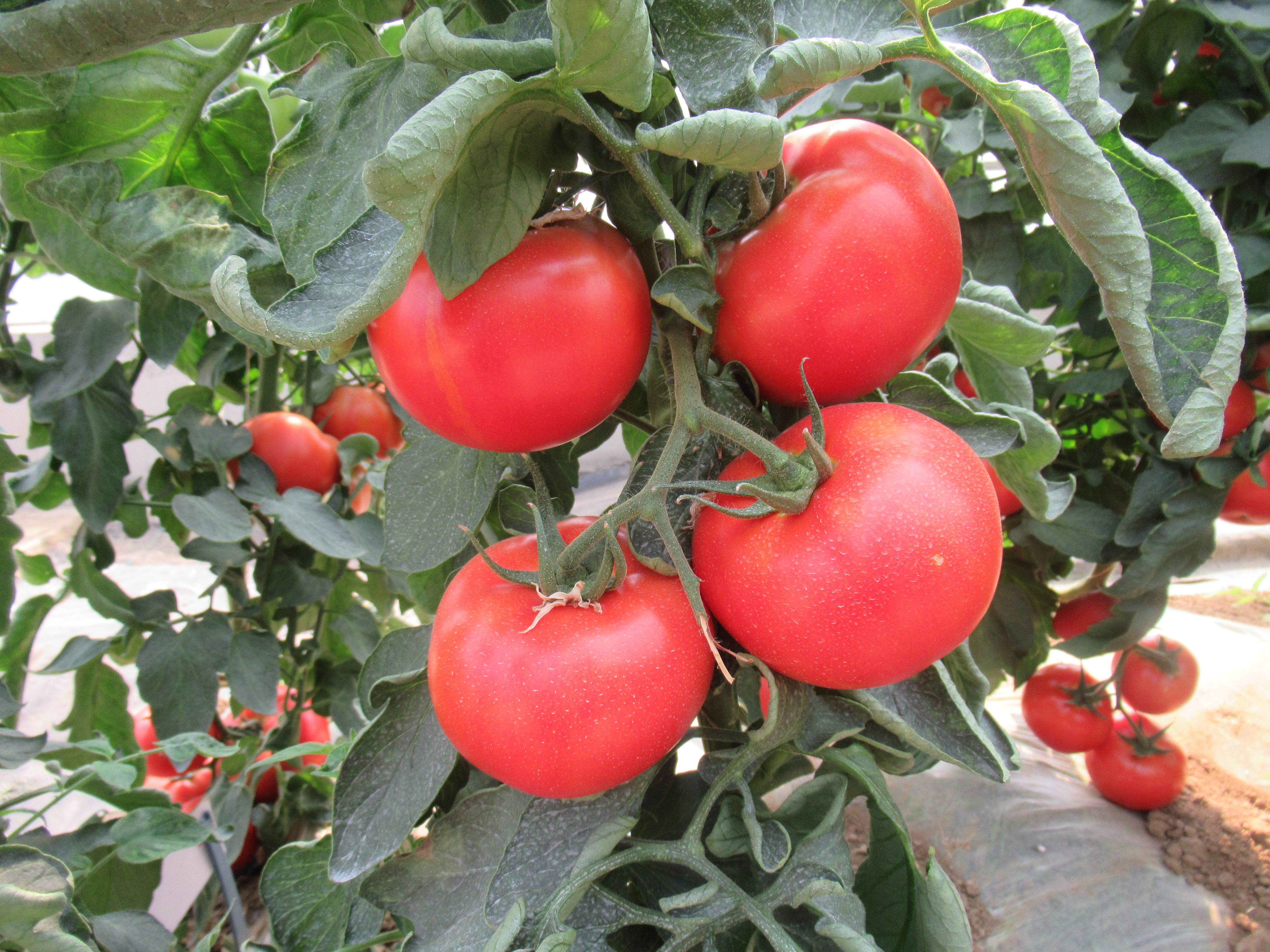

Supplement: Supplementary file 1 [file Data_Sheet_1.ZIP › Supplementary material/5.medium and large tomatoes.JPG]

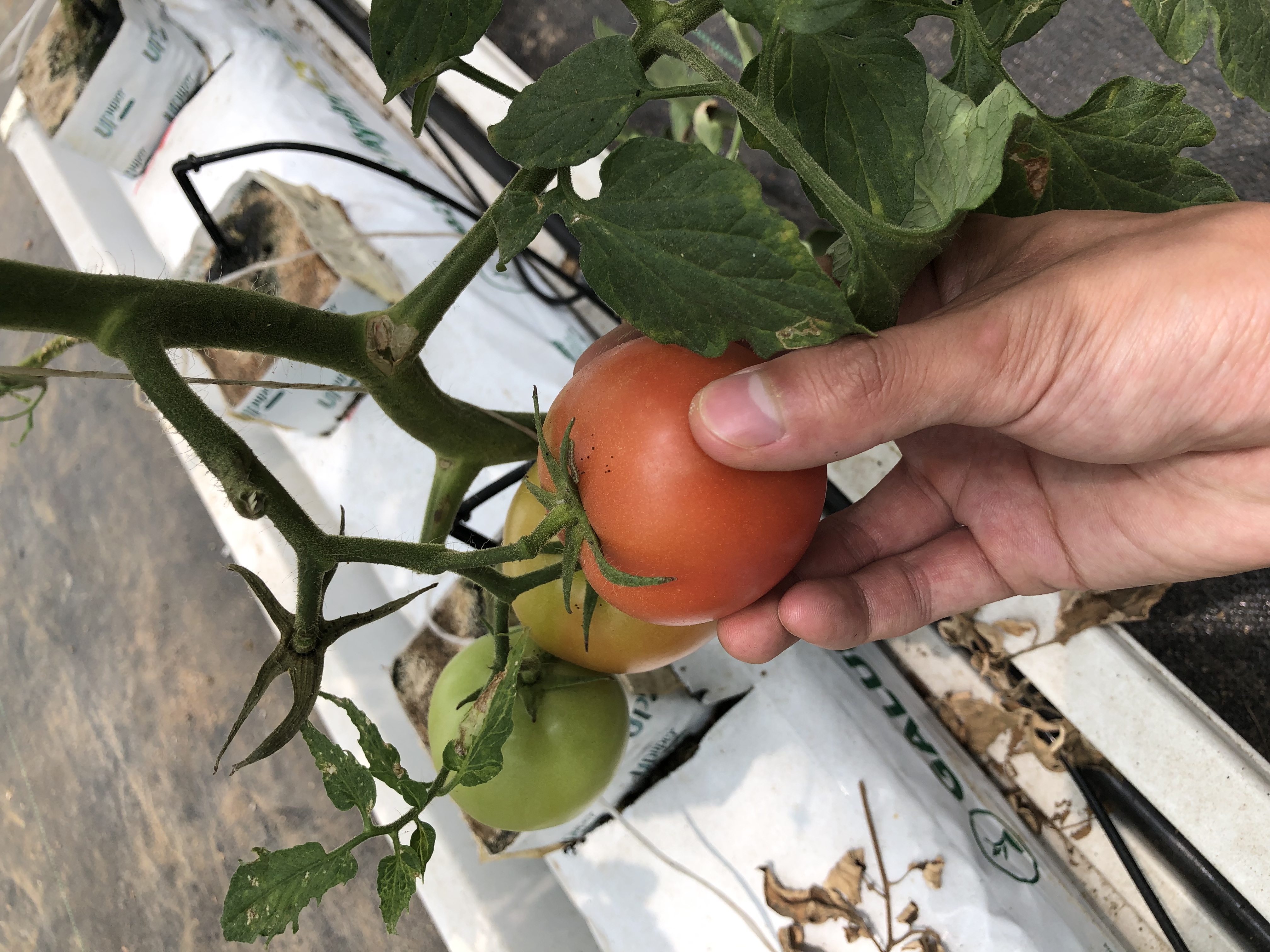

Supplement: Supplementary file 1 [file Data_Sheet_1.ZIP › Supplementary material/6. Fingertip pinch type.jpg]

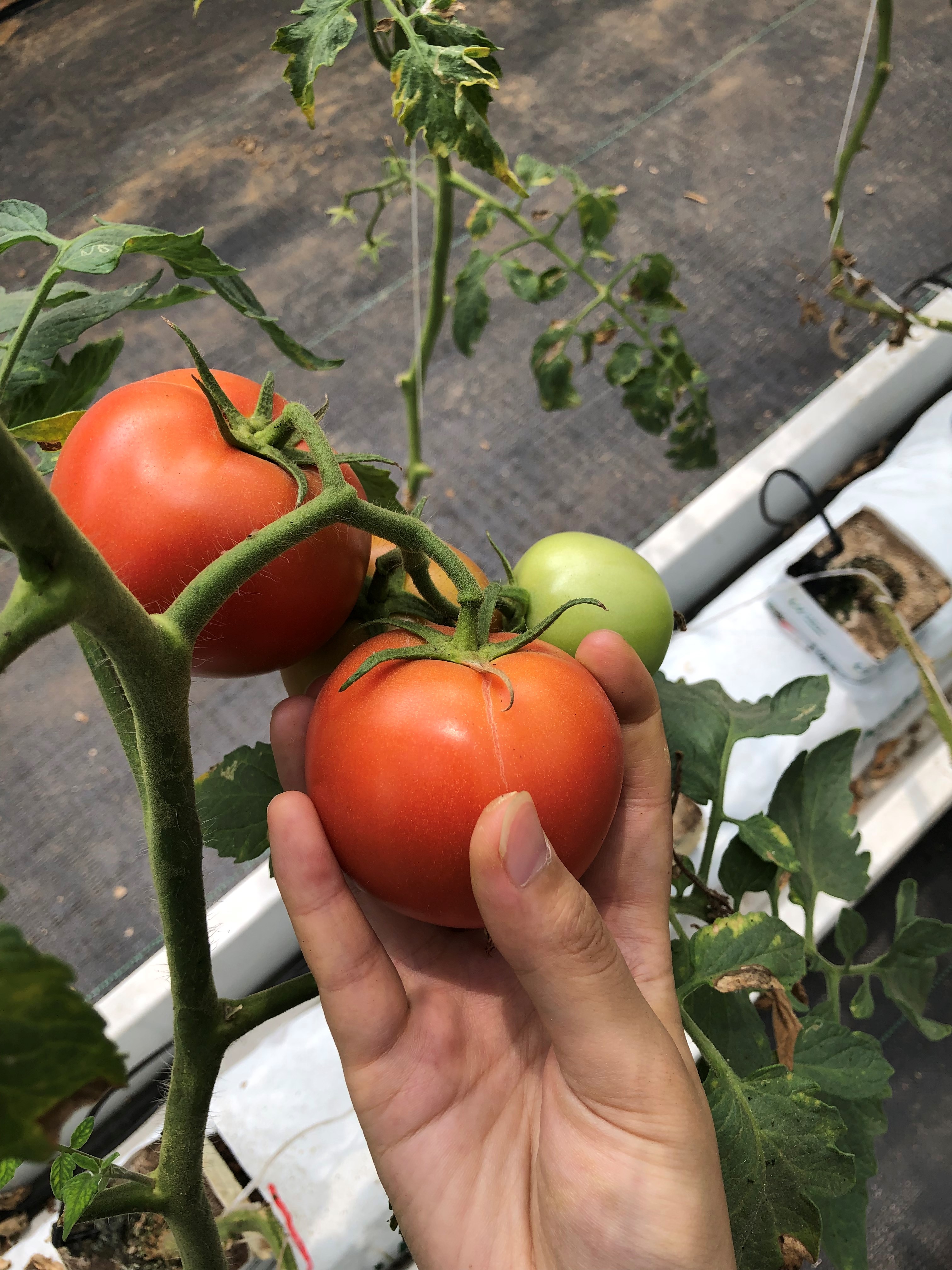

Supplement: Supplementary file 1 [file Data_Sheet_1.ZIP › Supplementary material/7. Semi-enclosed grip type for fingers and palms.jpg]

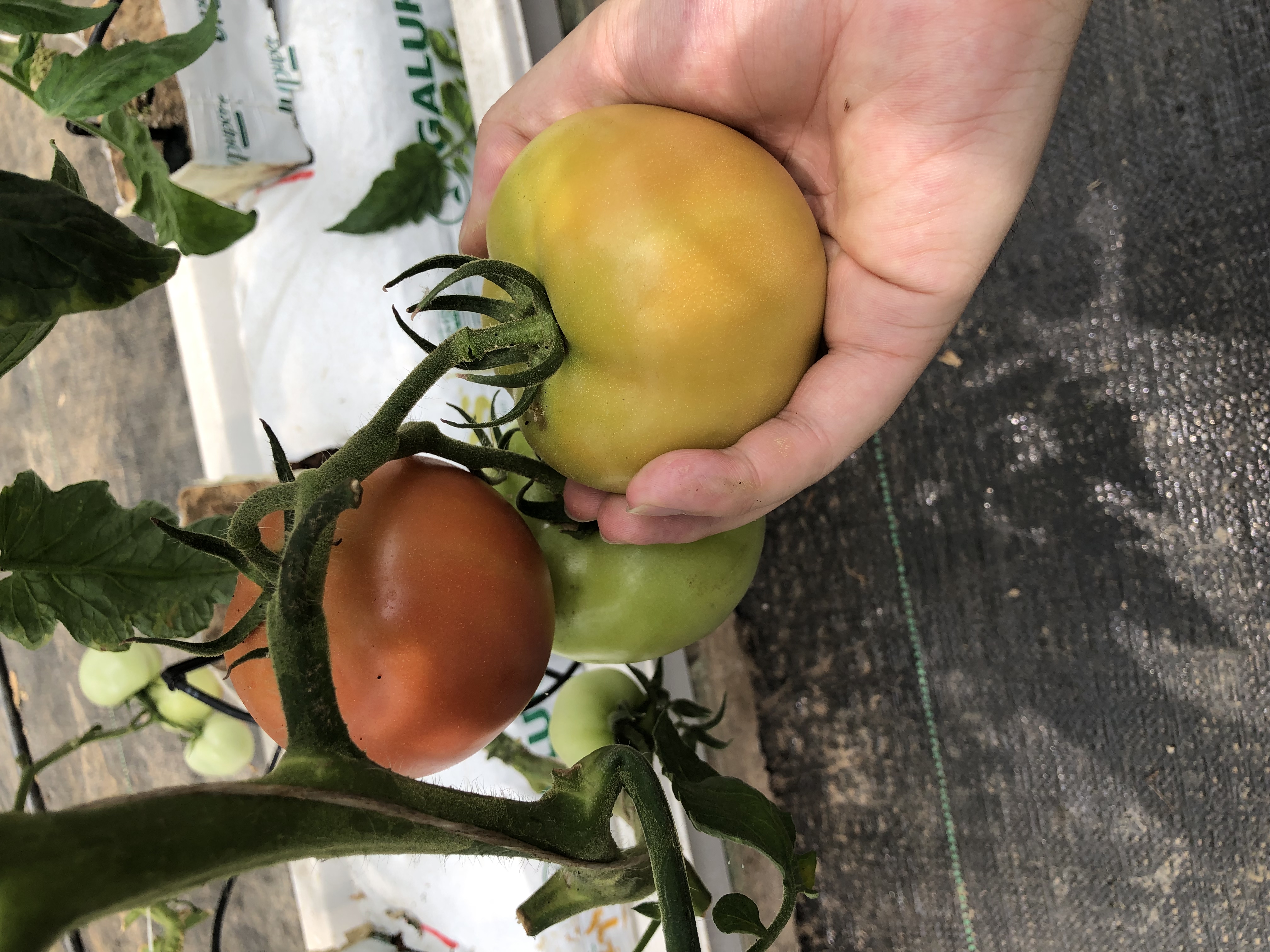

Supplement: Supplementary file 1 [file Data_Sheet_1.ZIP › Supplementary material/8. Full coverage grip type.jpg]
